# SOCIODEMOGRAPHIC DISADVANTAGE, LIVING WITH A SMOKER, AND OBESITY IN MIDDLE-AGED AND OLDER WOMEN

**DOI:** 10.1093/geroni/igz038.2553

**Published:** 2019-11-08

**Authors:** Carole K Holahan, Charles J Holahan, Sangdon Lim, Yen T Chen, Daniel A Powers

**Affiliations:** University of Texas at Austin, Austin, Texas, United States

## Abstract

Although sociodemographic disadvantage is a recognized risk factor for obesity, the potential role of living with a smoker in this relationship has been unexamined. This study investigated: (a) the association between sociodemographic disadvantage and living with a smoker, and (b) the role of living with a smoker in partially explaining the link between sociodemographic disadvantage and obesity. The study used limited access data from the Women’s Health Initiative Observational Study obtained from NHLBI. Participants were 91,888 women ranging in age from 50 to 79; 6,527 participants reported living with a smoker. Analyses were cross-sectional. Logistic regression analyses examined paths in the proposed model; bias-corrected bootstrapped confidence intervals tested indirect effects. All analyses controlled for age, marital status, and participants’ current smoking status. Results demonstrated a significant association (p < .001) between sociodemographic disadvantage and living with a smoker across three measures of disadvantage (for low education, low income, and Black ethnicity, ORs were 1.95, 2.10, and 2.63, respectively), as well as between living with and smoker and obesity (OR = 1.71). Moreover, the unstandardized indirect effect (CIs are in brackets) from sociodemographic disadvantage to obesity through living with a smoker was statistically significant for all three measures of disadvantage (for low education, low income, and Black ethnicity, indirect effects = .05 [.04, .06], .06 [.05, .06], and .07 [.06, .08], respectively). These findings underscore the need for innovative household-level interventions for disadvantaged families living with a smoker integrating smoking- and obesity-prevention efforts. This project was supported by the NIH/NCI (R03CA215947).

